# Heavy metals in Yinma River sediment in a major Phaeozems zone, Northeast China: Distribution, chemical fraction, contamination assessment and source apportionment

**DOI:** 10.1038/s41598-018-30197-z

**Published:** 2018-08-15

**Authors:** Jiunian Guan, Jia Wang, He Pan, Chen Yang, Jiao Qu, Nan Lu, Xing Yuan

**Affiliations:** 1 School of Environment, Northeast Normal University, Changchun, 130117 China; 2 College of Resources and Environment, Jilin Agricultural University, Changchun, 130118 China

## Abstract

Yinma River is a typical river in the major Phaeozems zone of Northeast China. It has been suffering an increasing environmental pressure from heavy metal contamination due to the rapid development of population, social-economy and urbanization as well as long term over cultivation. This study investigated the spatial distribution, chemical fraction of heavy metals (Cu, Pb, Zn, Cr, Cd, Ni, As, and Hg) in sediments of Yinma River based on BCR procedure, assessed the contamination level, and identified their sources via multivariate statistical analysis. The chemical fraction results indicated that Cd, Pb, Ni, and Zn exhibited higher mobility susceptibility and bioavailability with a significant and late anthropogenic origin. Hg and Cd might exert a potential hazardous influence on aquatic biota according to the geo-accumulation index (*I*_*geo*_). The pollution load index (*PLI*) assessment suggested that all of the sediment samples have been contaminated. Multivariate statistical analysis revealed that Zn, Cu, Hg, Cd, and Pb reflected the anthropogenic sources with a close correlation with TOC and socio-economic development; Ni, As and Cr tended to represent the geochemical background. Furthermore, Changchun City and Shitoukoumen Reservoir as the major drinking water source may be hotspots of the heavy metal contamination in the watershed.

## Introduction

Songhua River Basin is located in Northeast China, which is one of the three major zonal Phaeozems areas in the world, together with Mississippi River Valley in United States and Tineerbo River Basin in Ukraine. Yinma River is an essential tributary of Upper Songhua River, lying in the core area of the Phaeozems zone. Phaeozems is characterized by fertility, in particular with organic matter, such as humus^1^. Consequently, Yinma River watershed was established as a main grain production area and the major commodity grain base of rice, maize, and grain sorghum maize in China. The first heavy industrial base in China was also developed in this basin, with widely dispersed machine industry, chemical industry, power and manufacturing, metallurgical industry, grain processing industry, cement production, etc. Furthermore, it is also an extremely important water resource for the surrounding region, as Shitoukoumen Reservoir and Xinlicheng Reservoir are the major drinking water sources of Changchun City. Hence, the environmental quality of Yinma River is closely associated with public health, food and ecological security in the region. However, it has also been suffering an increasing environmental pressure from the rapid development of the population, social-economy and urbanization, together with long-term over cultivation. As a result, the water quality of Yinma River significantly has deteriorated lately, and it has been listed as the most polluted watershed in the Songhua fluvial system.

Rivers have an essential role in the acceptance and transportation of heavy metals, and river sediment serves as not only a major sink and carrier of heavy metals but also potential sources of secondary pollution, which can also reflect their contamination level^2,3^. Upper Songhua River was subjected to severe mercury pollution due to a large amount of discharge of raw chemical industrial sewage during the 1960s–1970s. Even though Hg concentration in the sediments decreased significantly after the 1970s, it still suffered moderate to extreme mercury pollution^4,5^. Lin *et al*. further investigated trace metal contamination in this river, indicating that the sediment was moderately contaminated by Cu, Pb, and Zn^6^. However, in the scope of our knowledge, there have hardly been any detailed studies on the heavy metal contamination in Yinma River watershed.

Heavy metals can induce various adverse environmental effects and severe environmental issues even though the water quality criteria are not exceeded, due to their properties of high toxicity, bioaccumulation and non-degradability and their ubiquitous nature^7,8^. In sediments, heavy metals are in existence in different chemical species that generally exhibit different environmental behaviours in terms of mobility, bioavailability and potential toxicity^9^. The chemical fractions can be operationally defined by a three-step sequential extraction procedure proposed by the European Community Bureau of Reference (BCR) as exchangeable, reducible, oxidizable and residual fractions^10^. It is generally accepted that the majority of heavy metals in non-polluted sediment exist in the residual fraction, which is unavailable for organisms, while the mobility of heavy metals can be indicated by the concentration of the exchangeable fraction, and the bioavailability is attributed to the sum of the exchangeable, reducible and oxidizable fractions^11^. Thus, it is necessary to investigate their distribution of chemical fractions in order to further reveal the environmental risks of heavy metals.

Additionally, total organic carbon (TOC) values of the sediments of aquatic environments in the Phaeozems zone were at a relatively high level, likewise Yinma River. Moreover, a continual inflow of soil has been brought forth due to serious catchment erosion induced by various anthropogenic activities, including deforestation, reclamation, and agricultural development, resulting in an enhancing accumulation of humic substances. Organic materials, especially humic substances, are regarded as a principal geo-sorbent for heavy metals^12^. Hence, TOC of the sediment is hypothesized to exert an important influence on the distribution of heavy metals. Furthermore, the distribution of heavy metals may vary widely and be characterized by the specific composition of pollutants in sediment and multiple pollution sources in the watershed of Yinma River, which are influenced by the combination of natural geological processes and anthropogenic activities^13^. In order to identify the influence of TOC and contribution sources of heavy metals, a multivariate statistical analysis was employed in the current study as an effective tool, including the methods of principal component analysis (PCA), hierarchical cluster analysis (HCA) and correlation analysis^14,15^.

The accumulation of heavy metals in the sediment poses a long term threat to the aquatic environments^16^. Consequently, there is a need for sediment quality indicators to assess the risks of contamination posed by heavy metals in aquatic environments. In recent decades, different sediment quality indicators have been developed, such as geo-accumulation index (*I*_*geo*_), which allows the practical determination of heavy metal concentrations for the examined sediment by comparing present concentrations with pre-industrial levels^17^. Therefore, it can exhibit an integrated picture of heavy metal contamination in the sediment, determining either the heavy metals in sediments presence as natural phenomena or anthropogenic activities^18^. However, *I*_*geo*_ focuses on contamination risk assessment of a single metal. Yet heavy metal pollution in aquatic environments generally occurs in the form of complex mixtures, which requires index reflecting the synergistic effects of its pollution^19^. The pollution load index (*PLI*) is established as the geometric mean of the contamination factor (*CF*) of each separate heavy metal in the examined sediment^20^. It provides a simple, comparative way for assessing the heavy metal contamination level and overall toxicity status of the sediment as a result of the contribution of all the examined heavy metals.

Therefore, based on chemical fractions and a multivariate statistical analysis, the primary objectives of the current study are to (i) investigate the concentrations and spatial distribution of heavy metals, including Cu, Pb, Zn, Cr, Cd, Ni, As, and Hg, in sediments of Yinma River; (ii) assess the bioavailability, enrichment, potential mobility, transferability, and contamination level of heavy metals in different chemical fractions in sediments via the BCR sequential extraction procedure, together with the geochemical indices of *I*_*geo*_, *CF*, and *PLI*; and (iii) identify the contribution sources of heavy metals by a multivariate statistical analysis including PCA, HCA and correlation analysis. The results provide basic information for the assessment of heavy metal environmental and ecological risks, the identification of anthropogenic influences, and fluvial utilization and supervision in typical rivers of Phaeozems zones.

## Materials and Methods

### Study area

**Figure 1:**
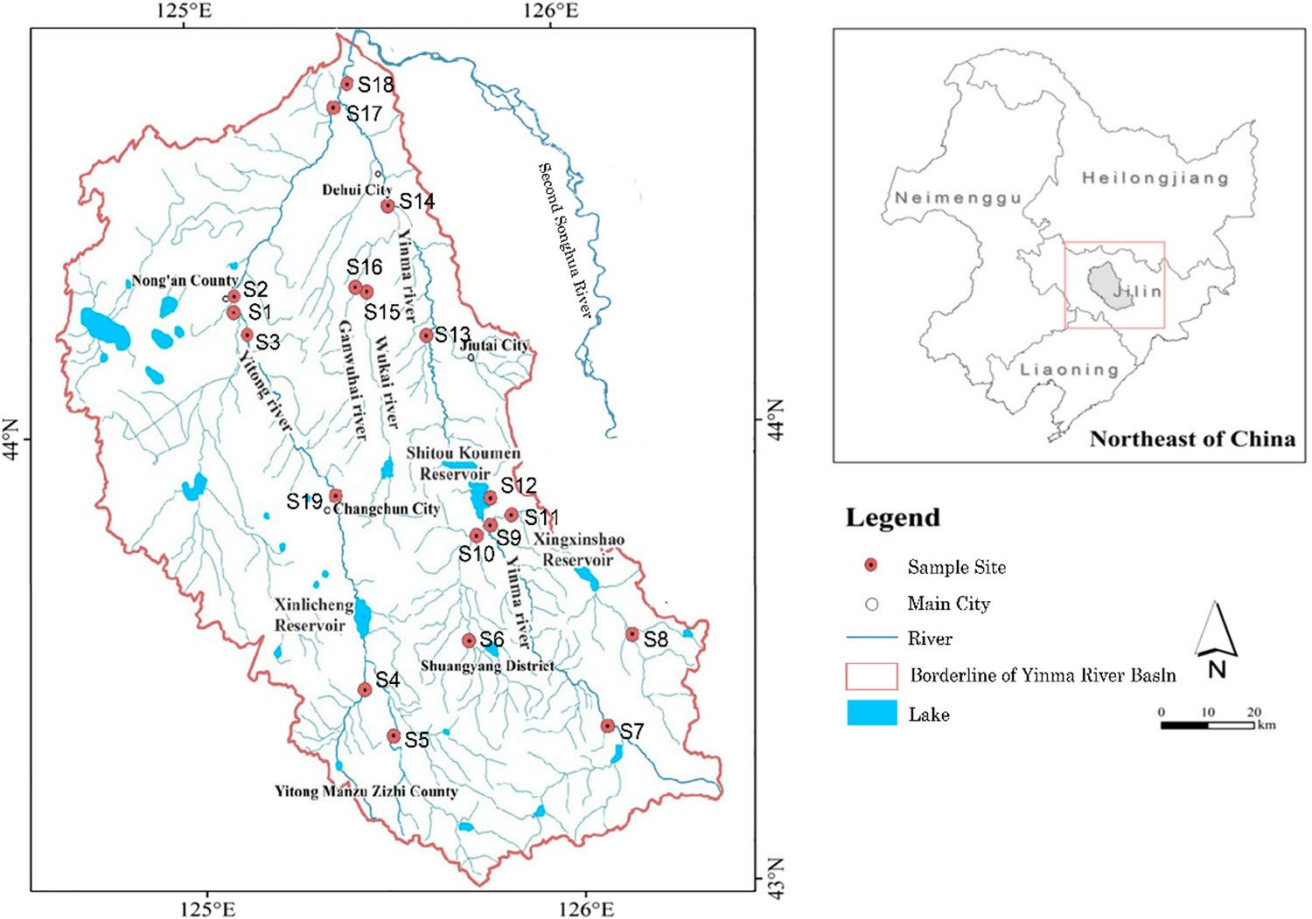
Study area of Yinma River and sampling sites location.

Yinma River (124°58′–126°24′E, 43°02′–44°53′N), located in central Jilin Province, is an essential tributary of Songhua River fluvial system (Fig. 1), with a total watershed area of 1.74 × 10^4^ km^2^ in which the plain areas accounts for 60%, and the rest is mountainous regions (Fig. S1). The watershed is mainly surrounded by the two major cities of Changchun and Jilin, which are the economic centres and important industrial bases in Northeast China. This watershed consists of two major rivers, i.e., Yinma River and Yitong River, in which Yitong River accounted for half of the total watershed area. The river originates in Hulan Ridge of Panshi City, merges with Yitong River in Nong’an County, and flows into the Second Songhua River in Kaoshan Town, with a length of 386.8 km. The watershed lies within a typical north temperate zone, with a continental monsoon climate. The average annual temperature is 5.3 °C with a frozen period of approximately 150 days. The annual precipitation ranges from 370 mm to 668 mm with an average of 587 mm.

The river flows through both Shitoukoumen Reservoir (125°43′–125°51′E, 43°47′–43°57′N) and Xinlicheng Reservoir (125°43′–125°50′E, 43°51′–43°57′N), which supply urban domestic water for Changchun City as well as agricultural irrigation water and fish farm water in the surrounding region. The total storage capacity is 1.28 × 10^9^ m^3^ for Shitoukoumen Reservoir and 5.92 × 10^8^ m^3^ for Xinlicheng Reservoir. The water supply of Shitoukoumen Reservoir was 392 million m^3^ in 2016, providing over 80% of the potable water for the metropolitan area (i.e., 3.5 million citizens) in Changchun, while Xinlicheng Reservoir accounts for 30% of the urban water supply.

### Sample collection

Surface sediment samples (0–20 cm) were collected from 19 sites along Yinma River and its tributaries during different seasons in 2016, i.e., pre-monsoon season (May), monsoon season (August), and post-monsoon season (October). Samples that exhibited no evidence of surface disturbance were retained. The sediments were collected using plastic grabs and transferred to pre-cleaned polythene sampling bags and sealed. Samples were transported to the laboratory in a portable refrigerator (0–4 °C) and kept at −4 °C in the laboratory before analysis processing. A Global Positioning System (GPS) was employed to locate the sampling sites.

### Sample preparation and analysis

The sediment samples were freeze-dried, crushed in an agate mortar, passed through a nylon 100-mesh sieve (<0.15 mm) and stored in pre-cleaned polythene bags at 4 °C in the dark before analysis of the properties and concentrations of heavy metals. The concentrations of heavy metals and chemical fractions of the sediment samples were measured in the Analytical Laboratory of the Northeast Institute of Geography and Agroecology, Chinese Academy of Sciences, which is certificated by China Metrology Accreditation by an inductively coupled plasma-mass spectrometer (Agilent 7500, USA). The chemical fractions of heavy metals were determined according to the optimized BCR three-step sequential extraction procedure^21^.

Laboratory quality assurance and quality control were assessed through the implementation of methods including analysis of triplicate samples, method blanks, and standard reference materials GBW07436. Analytical blanks and standard reference materials were run in the same way as samples. Sediment reference material was used to ensure the validation of data and the accuracy and precision of the analytical method (N = 3). The recoveries were 89.90–110.4% for all of the metals. The total concentrations of heavy metals were expressed as average values in mg/kg dry sediment. The differences in metal concentrations between analysed and certified values were generally <10%. Blank samples for the current methods were evaluated with each set of samples (N = 3). The relative deviation of the triple parallel samples was <5% in the batch treatments. All of the reagents were of analytical grade, and all of the solutions were prepared by ultra-pure water. All of the containers for the heavy metal analysis were soaked in HNO_3_ (10%) for at least 24 h and rinsed repeatedly with ultra-pure water.

### Assessment of heavy metal pollution in sediment

The determination of the pollution degree by a given heavy metal commonly requires both the metal concentration and its geochemical background values acting as reference baselines. Pollution, in this case, is assessed as the amount (or ratio) of the sample metal enrichment above the background values. Accordingly, the degree of heavy metal contamination in sediment is assessed by *I*_*geo*_, *CF*, and *PLI* in the current study.

#### Geo-accumulation index

*I*_*geo*_ has been widely applied to the assessment of soil and sediment contamination, which is defined as the following equation^17^:

1 $${{I}}_{{geo}}={\mathrm{log}}_{{\rm{2}}}(\frac{{{C}}_{{n}}}{{\rm{1.5}}{{B}}_{{n}}})$$

where *C*_*n*_ is the measured concentration of heavy metal (*n*) assayed in sediment samples and *B*_*n*_ is the geochemical background value of heavy metal (*n*). The value of 1.5 is introduced to minimize the possible variations in the background values due to lithogenic effects. A scale of *I*_*geo*_ indicates differences in the contamination level where it denotes uncontaminated sediments when *I*_*geo*_ ≤ 0 (class 1); uncontaminated to moderately contaminated when 0 < *I*_*geo*_ ≤ 1 (class 2); moderately contaminated when 1 < *I*_*geo*_ ≤ 2 (class 3); moderately to heavily contaminated when 2 < *I*_*ge*o_ ≤ 3 (class 4); heavily contaminated when 3 < *I*_*geo*_ ≤ 4 (class 5); heavily to extremely contaminated when 4 < *I*_*geo*_ ≤ 5 (class 6); and extremely contaminated when *I*_*geo*_ > 5 (class 7).

#### Contamination factor and pollution load index

The index *CF* is the ratio of the measured concentration of a given metal in the sediment to its background value, which has been classified into four grades as low contamination (*CF* < 1), moderate contamination (1 ≤ *CF* < 3), considerable contamination (3 ≤ *CF* < 6), and very high contamination (*CF* ≥ 6).

Whereas *PLI* provides a simple, comparative way to assess the level of heavy metal pollution, determined as the *n*^th^ root of the product of *n CF*:

2 $$PLI={(\prod _{{\rm{i}}}^{{\rm{n}}}{C}{{F}}_{{\rm{i}}})}^{{\rm{1}}/{\rm{n}}}$$

*PLI* > 1 suggests that pollution exists; otherwise, there is no pollution^20^.

### Multivariate statistical analysis

Multivariate statistical analysis was employed to deal with multiple-parameter issues on a series of heavy metals and relevant parameters. As the most widely used multivariate statistical method, PCA is a valid approach to investigate the major pollutants or identify the natural or anthropogenic pollution sources based on exploring the relationships and similarity of their distribution via extracting a reduced number of latent factors (principal components, PCs)^22^. Furthermore, all variables (heavy metals concentrations, pH, TOC, GDP, population and arable land amount) in the current study, were incorporated by HCA in order to identify different geochemical associations, and further provide details to verify the results of PCA. Moreover, HCA was also employed to group the sampling sites. Cluster analysis was performed on z-score transformed dataset to decrease the dimensionality of the dataset, and the squared Euclidean distance was applied to measure the distance between clusters of similar parameters^21,23^. Two-way HCA heat map was formed by the R statistical package (R version 3.1.3). Correlation analysis was also employed to identify the relationship among different heavy metals as well as other relevant parameters. Multivariate statistical analysis was performed using SPSS V23.0 software for Windows (IBM Corporation, Armonk, NY, USA).

## Results and Discussion

### Spatial distribution of heavy metals

**Figure 2:**
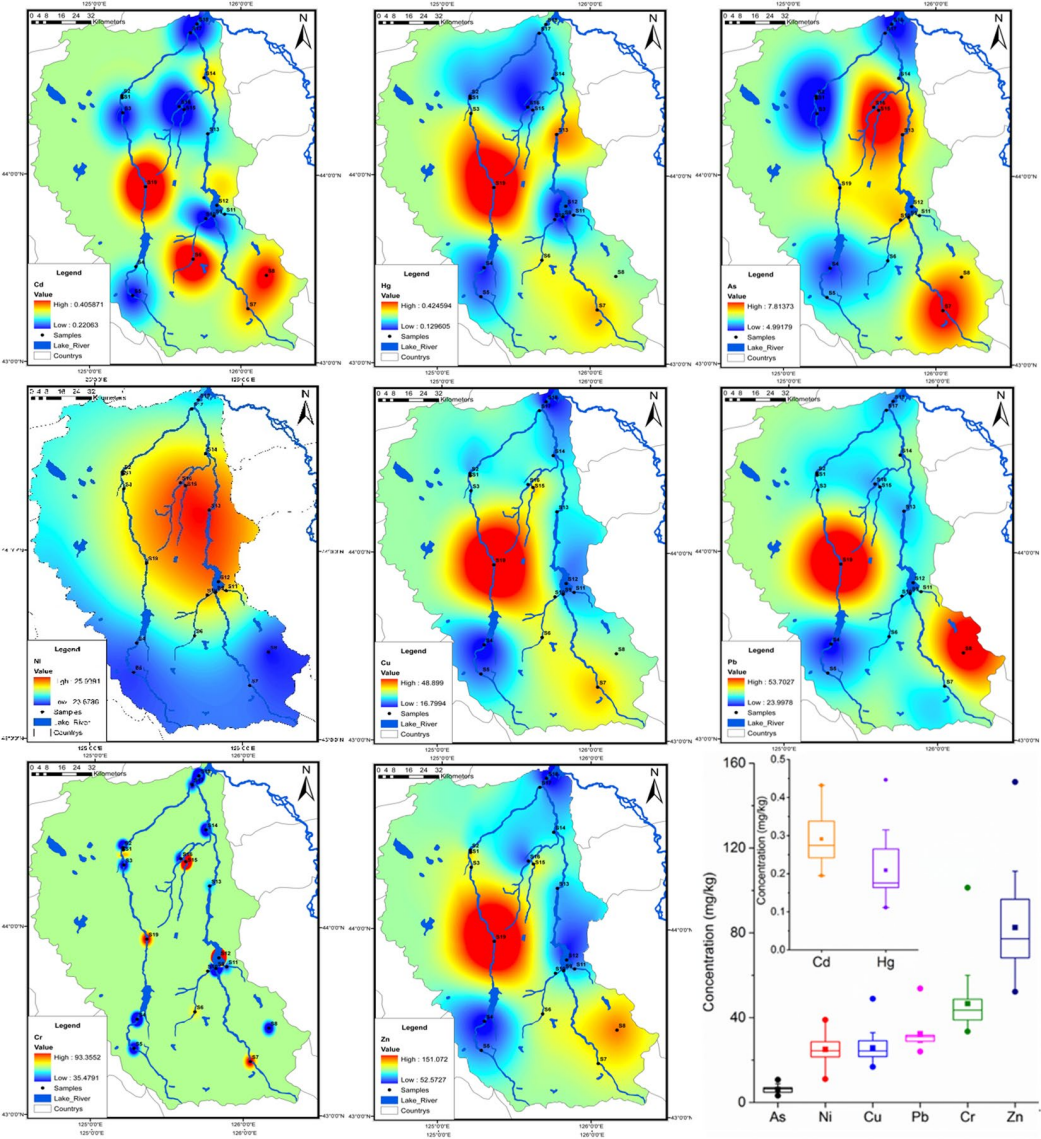
Spatial distribution of heavy metals in sediments.

The distribution of total heavy metal concentrations (Cd, Hg, As, Ni, Cu, Pb, Cr, and Zn) fluctuated widely in Yinma River Basin, following the order: Zn > Cr > Pb > Cu > Ni > As > Hg > Cd (Fig. 2). The concentrations of Zn in sediments varied from 52.23 mg/kg to 151.15 mg/kg, followed by Cr with a mean value of 46.60 mg/kg. The average concentrations for Pb, Cu, Ni, As, Hg and Cd were 32.38 mg/kg, 23.80 mg/kg, 25.06 mg/kg, 6.19 mg/kg, 0.21 mg/kg, and 0.29 mg/kg, respectively. The average concentrations of all the heavy metals exceeded their respective background values (Table S1), except for As.

The spatial distribution of heavy metals implied that the concentrations of Hg, Cu, and Zn peaked at Changchun City (S19), while the content of Cd reached its maximum levels in the tributaries of Shuangyang River (S6) and Chalu River (S8) in addition to Changchun City (S19). Pb content peaked at Changchun City (S19) and Chalu River (S8). As concentration peaked upstream of the main stream of Yinma River (S7) together with Wukai River (S15) and Ganwuhai River (S16) in the middle and downstream. The highest concentrations of Ni appeared at the middle reach of Yinma River (S13), including Shitoukoumen Reservoir (S12), Wukai River (S15) and Ganwuhai River (S16). The distribution of Cr was observed to be independent from each other, and the maximum value occurred at Shitoukoumen Reservoir (S12).

It is worth noting that the metals of Cd, Pb, Cr, Cu, Zn, and Ni were in comparably high concentrations at Shitoukou Reservoir and its upper tributaries; therefore, the potential risk of drinking water security cannot be ignored. Furthermore, higher concentrations of heavy metals were observed at both the upstream and midstream of Yinma River, which may exert potential influences on the water quality of lower reaches.

### Chemical fractions of heavy metals

**Figure 3:**
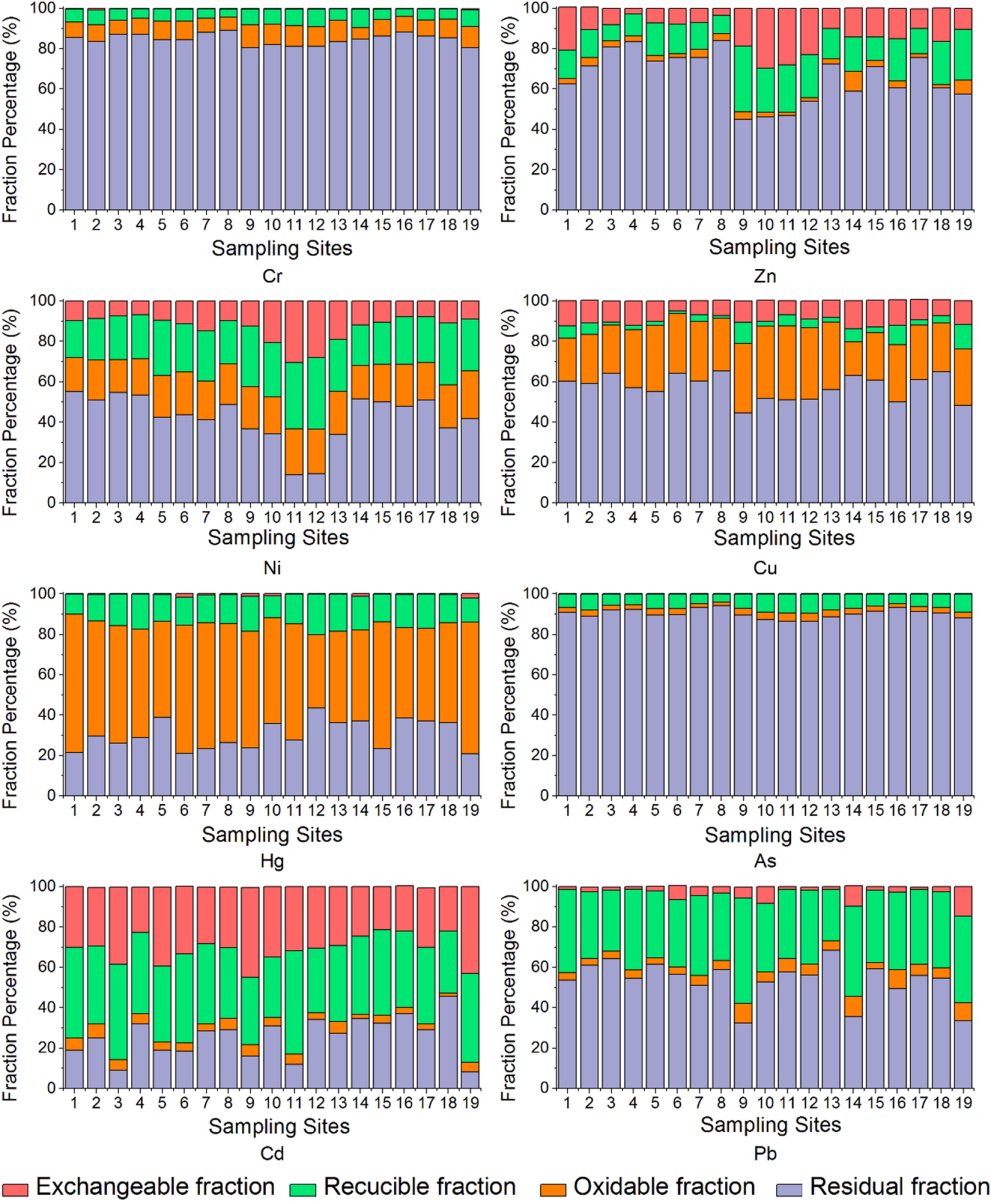
Chemical fractions of heavy metals in the sediment samples.

The results of chemical fractions for Cr, Zn, Ni, Cu, Hg, As, Cd, and Pb in sediments are demonstrated in Fig. 3. It is obvious that the distribution pattern greatly differed due to various factors.

Zn is a metal with relatively high mobility. In the current study, a large amount of Zn was associated with silicates as a residual fraction ranging from 44.83% to 84.02%, which is unlikely to release via dissociation. The generally low levels of exchangeable Zn may be attributed to the fact that it is readily absorbed by clays or hydrous metal oxides, and it is easily utilized by organisms in aquatic ecosystems^24^. However, the proportion of exchangeable Zn exceeded 20% at S1, S10, S11, and S12, which may be attributed to its wide application as an effective ingredient of bactericide and fertilizer in agricultural production. Additionally, Zn is commonly added to livestock feed due to its growth-promoting and antibiotic attributes^25^. Therefore, the livestock breeding sewage may also largely contribute to Zn contamination in sediment.

Although Cr represented a high concentration level in the sediment, the majority of it was associated to the residual fraction fluctuating from 80.58% to 89.20%. No more than 0.94% was observed in the exchangeable fraction, indicating a total insignificance in geochemistry. The average proportions of the oxidizable and reducible fractions were 8.64% and 6.28%, respectively. As a kind of highly toxic heavy metal, Cr has been proven to be a carcinogenic substance, which is toxic to aquatic organisms even at low concentrations.

Apart from the residual fraction (32.41%–68.42%, average of 53.53%), Pb was dominated by the reducible fraction (25.37%–52.04%, average of 37.24%), which is attributed to the high adsorption capacity of Pb by Fe/Mn oxyhydroxides^26^. However, as a highly toxic heavy metal, Pb is potentially hazardous to aquatic biota, since it can be remobilized due to variations in physicochemical and biological conditions. Moreover, the exchangeable Pb percentage exceeded 10% at the typical urban areas in the watershed at S14 and S19, indicating a recent introduction from external sources like anthropogenic activities, which may exert an adverse influence on aquatic biota^27^.

Likewise, most of Cu was also in association with the residual fraction, with an average value of 59.62%. The oxidizable fraction (16.71%–36.77%, mean of 28.44%) was the predominant form in all of the sediment samples, which is strongly composed of organics with a high stability constant. Desorption from organic materials generally requires a high activation energy to overcome^28^. In addition, a portion of exchangeable Cu was observed representing 5.05%–14.08%, with a mean of 10.07% of the total. Cu also acts as a commercial fertilizer and pesticides, which may explain its existence in fractions to a certain extent^29^.

Ni can be introduced into rivers by natural rocks as well as anthropogenic activities, including mining, smelting, refining, and alloy processing^30^. Although Ni is essential for the growth of diverse microorganisms, plants and several species of vertebrates, its compounds were classified as human carcinogens^31^. In the current study, the proportion of residual Ni in sediment (13.81–55.02%) was at a relatively low level in comparison with other metals. However, the exchangeable Ni showed the opposite pattern (6.79–30.46%), in particular with S11 and S12, the residual fraction accounted for only 13.81% and 14.39%, respectively, while the exchangeable fraction was 30.46% and 27.98%, respectively. An appreciable percentage of Ni was observed in the reducible fraction, representing 18.16–35.55% of the total content. This pattern may be attributed to the high Ni reserves in the watershed and the relevant industrial activities, such as mining, smelting, and combustion of fuel^32^. For example, one of the largest nickel industry companies in China is located in Panshi City.

As is primarily bound to the residual fraction with an average percentage over 90%, demonstrating that the majority of As was likely to be incorporated with aluminosilicate minerals, displaying relatively limited mobility and bioavailability.

The contamination of Hg is attracting the most concern among different heavy metals due to the extremely high toxicity and biomagnification rate in the food chain^33^. Unlike other heavy metals, most of Hg in the sediments (36.40–68.67%, average of 54.47%) was in the oxidizable fraction, which agreed with the fact that the distribution of Hg is governed by high-molecular-weight organic matter coordinated with sulphur ligands in sediments^34^. The proportion of exchangeable Hg was averaged at 0.57%, suggesting that there were limited recent inputs. Consequently, the retention and bioavailability of Hg may largely depend on the content and composition of organic materials in the sediments.

Cd is one of the most toxic heavy metals threatening aquatic environments. In the current study, the predominant proportions of Cd were illustrated as the reducible fraction (29.91% to 51.29%) and the exchangeable fraction (21.39% to 44.62%). In particular, the percentages of exchangeable Cd in S9 and S19 were over 40% of the total, indicating a greater degree of mobility and bioavailability. Fe/Mn oxyhydroxides have been documented to represent a significant adsorption capability of Cd due to their complex structures with poor crystallinity^35^. The presence of exchangeable Cd may be attributed to the recent input via anthropogenic activities, such as the smelting of non-ferrous metals, the pharmaceutical industry, electrical appliances industry, road traffic (wear of tires), as well as fertilizer application in agricultural production^36^. Besides, the similarity of the ionic radius of Cd and Ca may also accelerate the co-precipitation and incorporation of Cd with carbonates such as calcite lattice^37^.

The results of chemical fractions illustrated that even though considerable proportions of heavy metals were in the residual fraction, the toxic metals of Cd, Ni, Pb, and Zn may exert greater potential hazardous influence, due to their higher susceptibility, migration capability, and bioavailability with strong anthropogenic sources in the current basin. The recent input of heavy metals initially exists in the unstable chemical fractions such as exchangeable and reducible fractions^38^. On the contrary, Cr and As exhibited considerably low proportions in exchangeable and reducible fractions, demonstrating a rather limited availability in all of the sediment samples. They may mainly be controlled by lithology^39^. The reducible Pb, Cd, and Ni dominated the bioavailable fractions in all of the sedimentary samples, which may be ascribable to the fact that Fe and Mn oxyhydroxides can scavenge them via adsorption, flocculation and co-precipitation in aquatic systems and further generate stable complexes^40^. Cu and Hg exhibited higher proportions in the oxidizable fraction, since they are affinitive to organic substances in the sediment and strongly complexed with humic substances. Furthermore, the metals of Cd, Pb, Ni, and Zn were in comparably reactive fractions, i.e., exchangeable and reducible fractions at Shitoukoumen Reservoir and its upper tributaries (S9–S12), which may have adverse impacts on the drinking water security.

### Heavy metals pollution assessment

**Figure 4:**
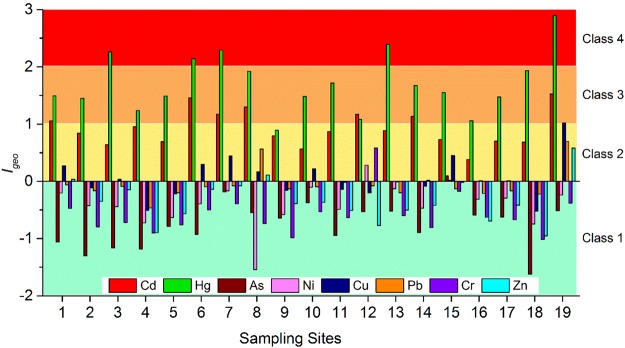
*I*_*geo*_ values for heavy metals at different sampling sites.

The degree of heavy metal pollution was categorized into different classes according to the values of *I*_*geo*_ which fluctuated from −1.62 (As at S18) to 2.90 (Hg at S19) (Fig. 4). Class 4 refers to “moderately to heavily polluted” consisting of only Hg (S3, S6, S7, S13, and S19). Class 3 refers to “moderately polluted” including the metals of Hg (S1, S2, S4, S5, S8, S10–S12, and S14–S18) and Cd (S1, S6–S8, S12, S14, and S19). While Cd (S2–S5, S9–S11, S13, and S15–S18), Hg (S9), As (S15), Ni (S12 and S15), Cu (S1, S3, S6–S8, S10, and S15–S17), Pb (S8, S14, and S19), Cr (S12), and Zn (S1, S8, and S19) were ranked as unpolluted to moderately polluted level (Class 2). The rest of the sites were practically not under pollution of the heavy metal (Class 1).

The result of *I*_*geo*_ indicated that the sediment was mainly impacted by Hg and Cd. The thermopower plants may largely contribute to the Hg and Cd contamination in the sediment, especially in urban areas^41^. The coal-fired power plant was revealed to be the main contributor to anthropogenic sources of both Hg and Cd in China^42,43^. In the current watershed, there are approximately 10 large-scale thermopower plants with a long period of heating, usually lasting nearly six months. Cement plants, municipal solid waste incinerators and vehicle emissions may also largely contribute to the Hg contamination^44^. Automobile and cement production forms the backbone of industry in the research area, with an annual vehicle output of over 2.5 million and over 20 million tons of cement. There are approximately 2.86 million civilian vehicles in Changchun City; therefore, cement production and vehicle emission may also be important contributors to Hg contamination. Agricultural production could be described as a key source of Cd emissions, since it indicates the agricultural activities of fertilizer and pesticide application in the surrounding countryside where most phosphate fertilizers contain Cd^45^.

**Figure 5:**
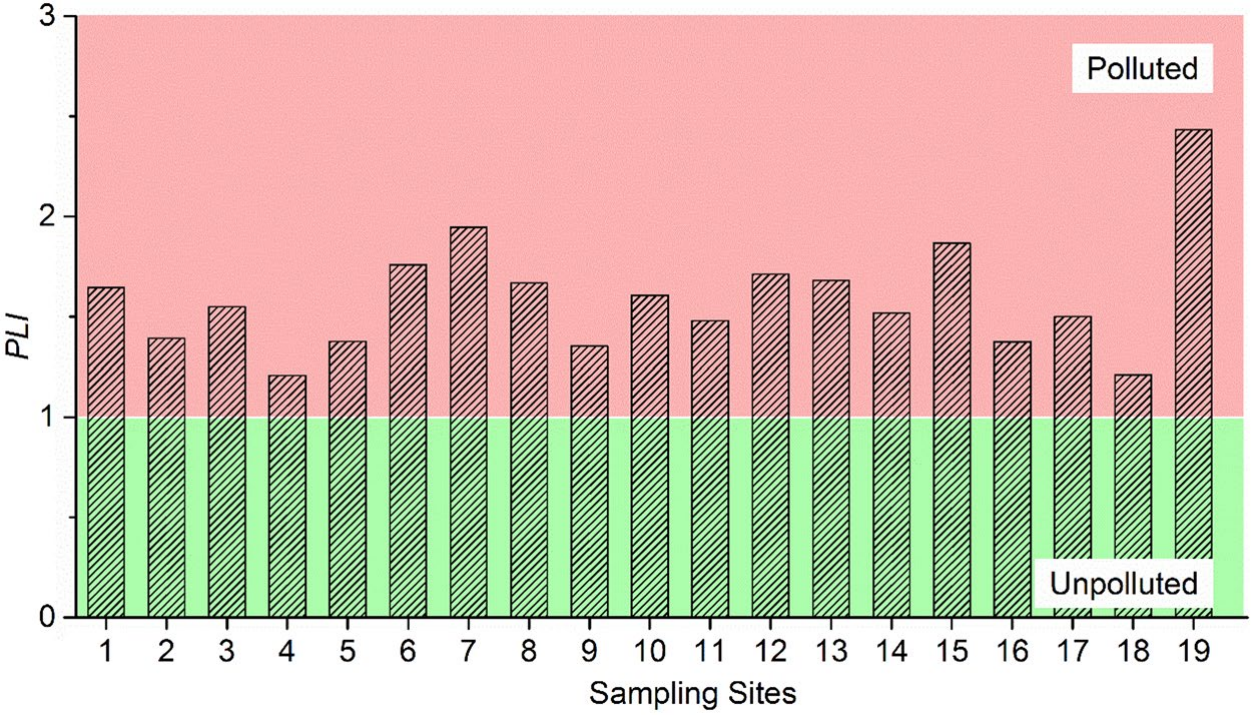
*PLI* values for heavy metals at different sampling sites.

Heavy metals with high *CF* values normally possess low retention times and high mobility in sediment, which pose high risks to the aquatic environment and ecosystem^16^. According to the contamination categories of *CF*, most heavy metals exhibited low to moderate pollution along the river, with the exception of Cu, Cd and Hg (Fig. S2). There was considerable Cu contamination at S19, and Cd contamination at S1, S6, S7, S8, S12, S14, and S19. There was considerable Hg contamination at all of the sampling sites for Hg, apart from S3, S6, S7, S13, and S19 (very high contamination) and S9 (moderate contamination). *PLI* may provide a certain understanding of the quality of the aquatic environment, together with valuable information on the pollution status of the aquatic system^46^. The values of *PLI* fluctuated from 1.21 to 2.37, suggesting that all of the sediment samples have been polluted by heavy metals (Fig. 5). In addition, the values peaked at S19, which was in agreement with the result of *I*_*geo*_.

### Multivariate statistical analysis

**Figure 6:**
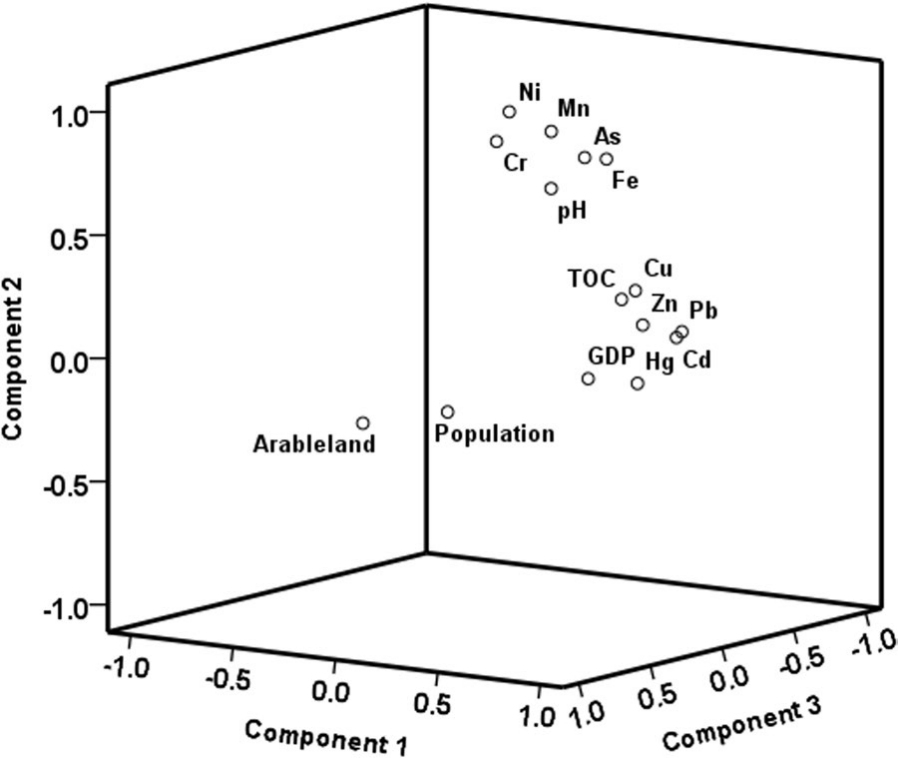
The principal component analysis loading plot for heavy metals and relevant parameters.

The results of PCA revealed that there were three principal components (PCs) in the samples explaining 86.22% of the total variance, which was sufficient to provide an acceptable sight of the data structure (Fig. 6) and the results of Kaiser-Meyer-Olkin and Bartlett’s tests were exhibited in Table S2. PC1 contributed 45.15% to the total variance, which was composed of more significant factors than the others and characterized by high loadings of Zn (*r* = 0.89), Cu (*r* = 0.84), Hg (*r* = 0.81), Cd (*r* = 0.77), Pb (*r* = 0.68), TOC (*r* = 0.82), and GDP (*r* = 0.77). The metals in PC1 tended to closely associate with anthropogenic activities including agricultural practices (such as the application of fertilizers, pesticides and livestock breeding), industrial activities (such as metallurgy), and the combustion of fossil fuels (such as coal) as previously discussed. This result was also in agreement with the relatively high *I*_*geo*_ values of these metals. It was also observed that GDP had a significant correlation with the concentrations of Hg, Cu, Pb and Zn (Table S3), suggesting that the economic development may somehow contribute to the contamination of these metals. Thus, PC1 can reflect the contribution of anthropogenic sources of heavy metals.

Furthermore, the metals in PC1 were remarkably related to TOC of the sediments (Table S3). Organic materials are regarded as a vital factor in the geochemical behaviour and ecotoxicity of heavy metals^47^. In the current watershed, TOC in the sediment fluctuated from 1.06% to 7.10%, with a mean value of 3.25% (Fig. S3), peaked at Changchun City (S19). It has been documented that Yinma River is supplying tremendous TOC to the Songhua River, which is in accordance with the results that the values were higher than the reported TOC of sediments from the Songhua River (0.21–8.61%, mean 2.48%)^48^. Humic substance originating from Phaeozems dominates TOC in the sediment, which can reduce the mobility, bioavailability and toxicity of heavy metals via formation of stable organometal complexes^49^. Furthermore, the comparably high concentrations of both TOC and heavy metals in Changchun City may suggest that the anthropogenic organics may also exhibit a strong capability for heavy metal adsorption.

PC2 took into account 25.57% of the total variance, with significant loadings of Ni (*r* = 0.96), As (*r* = 0.75), Cr (*r* = 0.83), Fe (*r* = 0.81), Mn (*r* = 0.85) and pH (*r* = 0.72). Concentrations of Ni, As and Cr in the sediment samples were close to their background values, and *I*_*geo*_ values were lower than that of metals in PC1. Moreover, all of these heavy metals were strongly associated with Fe and Mn, which are commonly used as the geochemical reference element because the geochemistries of Fe and Mn are similar to other trace metals, and their natural concentrations tend to be uniform^50^. Therefore, PC2 reflects the contribution of natural geological sources.

Moreover, pH is an essential factor controlling the mobility of heavy metals in aquatic systems. High pH values ( > 6.5–7.0) can limit their solubility and bioavailability via promoting adsorption and precipitation^51^. Low pH will reduce the negative surface charge of clay particles, including organic matter and Fe/Mn/Al (hydro)oxides, especially soluble sulphides and increase the activity of cations such as H^+^, Fe^3+^, and Al^3+^ that will compete with heavy metal cations for negative sorption sites in sediments^52^. Hence, a decrease in pH can actually weaken the strength of association between heavy metals and sediment and further impede the retention of the metals. pH values in the current sediment samples varied from 6.21 to 8.65, with an average of 7.72 (Fig. S4). The high pH under different hydrological regimes may be interpreted by the alkaline geologic formations, which originated from the second geological era and runoff generation, indicating a relatively stable condition for heavy metals^6^. However, it is worth noting that even though the sediments were alkaline, the water column of Yinma River was in an acidic condition, with an average pH value of 6.23, which may facilitate the release of heavy metals from the sediment and induce secondary pollution.

PC3 represented 15.50% of the total variance in which the population and arable land amount accounted for a heavy loading (*r* = 0.88 and 0.91, respectively). There was no heavy metal associated with PC3.

**Figure 7:**
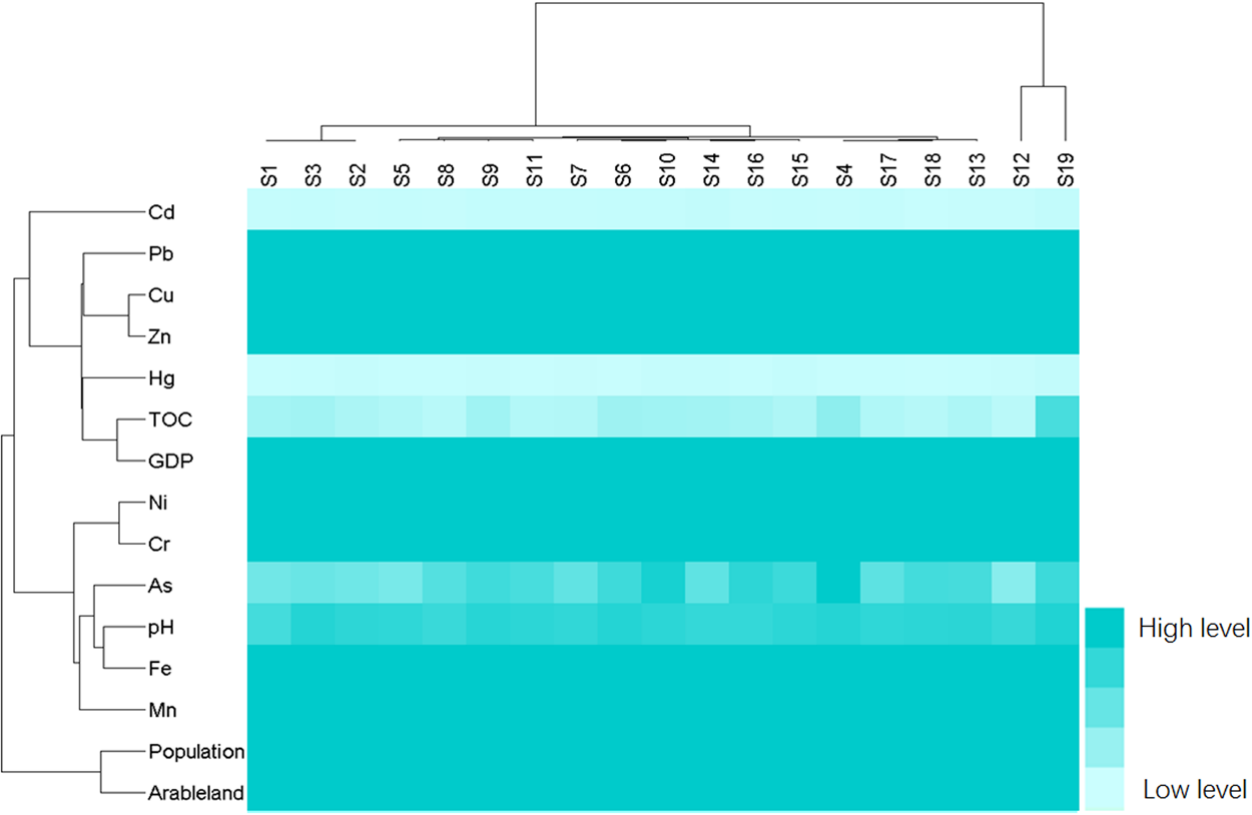
The results of hierarchical clustering analysis for heavy metals, sampling sites and other parameters.

The data were sorted using HCA to create the dual dendrogram (Fig. 7). The horizontal dendrogram exhibits HCA of sampling sites (S1–S19) according to different obtained variables related to the sampling sites. The sampling sites were separated into 3 clusters by a clustering analysis with a distance of 8. Cluster 1 consisted of the sites of S1–S11, and S13–S18, while S12 and S19 were listed as cluster 2 and cluster 3, respectively, which illustrated no similarity with other sampling sites. S19 represented Changchun City as the largest industrial city in Yinma River Basin. The concentrations of Cd, Hg, Cu, Pb and Zn, together with GDP and the population, peaked at S19, indicating a comparably heavy contamination and strong influence of anthropogenic activities. S12 refers to Shitoukoumen Reservoir, which not only functions as a drinking water resource but also supplies water for fishery, agriculture and recreational uses. The contents of Ni and Cr reached the maximum values in the reservoir, and the reservoir received a large quantity of heavy metals from the upstream brought forth by industrial, agricultural, and domestic sewage, as well as soil erosion. These heavy metals threaten the drinking water safety for the residents, which agreed with the results of the chemical fractions. The rest of the sites showed similarity with each other, to a certain extent.

The vertical dendrogram represents the results of the clustering analysis of all the parameters in the current study according to the mean Euclidean distances, which demonstrates that all of the parameters can be clustered into 3 different clusters. Cluster 1 consisted of Cu, Zn, Pb, Cd, Hg, TOC and GDP. Cluster 2 contained Ni, Cr, As, Fe, Mn and pH. The population and arable land amount are listed as cluster 3. Cluster 1 shows that the metals of Cu, Zn, Pb, Cd, and Hg may share similar geochemical behaviours and sources, which were significant for TOC and socio-economic development. Ni, Cr, As, Fe, Mn and pH were clustered in cluster 2. Ni, Cr and As were significantly correlated to both Fe and Mn, suggesting that they may tend to focus on the Fe and/or Mn oxyhydroxides, which was in line with the results of the correlation analysis (Table S3). Cluster 3 also represents the parameters of population and arable land amount. In conclusion, the results of HCA were consistent with the results of PCA.

## Conclusions

The chemical fraction of heavy metals and indices of *I*_*geo*_, *CF*, and *PLI*, together with multivariate analysis techniques, have been employed to evaluate sediment pollution in Yinma River. Even though a considerable proportion of heavy metals was in the residual fraction, the toxic metals of Cd, Ni, Pb, and Zn may exert greater potential hazardous influence with strong anthropogenic sources, in particular with Shitoukoumen Reservoir and its upper tributaries, which may threaten the drinking water security. Cu and Hg exhibited high affinities to organic substances in the sediment. The results of PCA and HCA indicated that the metals of Zn, Cu, Hg, Cd, and Pb were significantly correlated with the TOC of the sediments and/or socio-economic development, which may represent anthropogenic sources. Ni, As and Cr tended to reflect the geochemical background. Furthermore, Changchun City and Shitoukoumen Reservoir could be hotspots of heavy metal contamination in the watershed. Based on *I*_*geo*_ and *PLI* assessment, all of the sediment samples have been polluted with Hg and Cd. Therefore, heavy metals may exert potential hazardous influences on the eco-security in Yinma River, in particular within the urban areas and drinking water resources. Organic matter may be essential in the distribution of different heavy metals, including Cu, Hg, Cd, Pb, and Zn. The spatial temporal distribution of heavy metals as well as their temporal vertical distribution in the sediment should be should be further investigated in the future, together with mechanisms of the interaction between heavy metals and the major components in the sediment.

## Electronic supplementary material

Supplementary information
