# Supplementary material for: Heavy metals in Yinma River sediment in a major Phaeozems zone, Northeast China: Distribution, chemical fraction, contamination assessment and source apportionment
Source: Sci Rep. 2018 Aug 15;8:12231. doi: 10.1038/s41598-018-30197-z (PMC6093934; doi:10.1038/s41598-018-30197-z)
Supplement: Supplementary file 1 — Supplementary information [file 41598_2018_30197_MOESM1_ESM.docx]

**Heavy metals in Yinma River sediment in a major Phaeozems zone, Northeast China: Distribution, chemical fraction, contamination assessment and source apportionment**

Guan Jiunian^a†^, Wang Jia^a†^, Pan He^b^, Yang Chen^a^, Qu Jiao^a^, Lu Nan^a*^, Yuan Xing^a*^

^a^ School of Environment, Northeast Normal University, Changchun 130117, China

^b^ College of Resources and Environment, Jilin Agricultural University, Changchun 130118, China

**Caption of tables:**

Table S1. Background values of heavy metals in the Yinma River

Table S2. Kaiser-Meyer-Olkin and Bartlett’s tests

Table S3.Pearson correlation matrix of the parameters

Table S1. Background values of heavy metals in the Yinma River*

| Background values (mg/kg) | | | | | | | |
| --- | --- | --- | --- | --- | --- | --- | --- |
| Cd | Hg | As | Ni | Cu | Pb | Cr | Zn |
| 0.10 | 0.04 | 6.7 | 21.4 | 16.1 | 22.1 | 45.1 | 67.4 |

*: China Environmental Monitoring Station, 1990. Natural Background Values of Soil Elements in China. China Environmental Science Press, Beijing, China (in Chinese).

Table S2. Kaiser-Meyer-Olkin and Bartlett’s tests

| KMO Measure of Sampling Adequacy | | 0.631 |
| --- | --- | --- |
| Bartlett’s Test of Sphericity | Approx. Chi-Square | 3257.406 |
|  | *df* | 78 |
|  | Sig. | 0.000 |

Table S3. Pearson correlation matrix of the parameters

|  | Hg | As | Ni | Cu | Pb | Cr | Zn | Fe | Mn | pH | TOC | GDP | Population | ALA |
| --- | --- | --- | --- | --- | --- | --- | --- | --- | --- | --- | --- | --- | --- | --- |
| Cd | 0.56^**^ |  |  | 0.55^**^ | 0.61^**^ |  | 0.61^**^ | 0.52^*^ |  |  | 0.56^**^ |  |  | 0.48^*^ |
| Hg | 1 |  |  | 0.69^**^ | 0.48^*^ |  | 0.68^**^ | 0.46^*^ |  |  | 0.68^**^ | 0.73^**^ |  |  |
| As |  | 1 | 0.49^*^ | 0.52^*^ |  |  |  | 0.76^**^ | 0.73^**^ | 0.48^*^ |  |  |  |  |
| Ni |  |  | 1 |  |  | 0.77^**^ |  | 0.58^**^ | 0.47^*^ |  |  |  |  |  |
| Cu |  |  |  | 1 | 0.58^**^ |  | 0.92^**^ |  |  | 0.62^**^ | 0.93^**^ | 0.76^**^ |  |  |
| Pb |  |  |  |  | 1 |  | 0.73^**^ | 0.48^*^ |  | 0.56^**^ | 0.61^**^ | 0.47^*^ |  |  |
| Cr |  |  |  |  |  | 1 |  | 0.62^**^ | 0.56^*^ | 0.50^*^ |  |  |  |  |
| Zn |  |  |  |  |  |  | 1 | 0.56^**^ |  | 0.63^**^ | 0.86^**^ | 0.68^**^ | 0.54^*^ |  |
| Fe |  |  |  |  |  |  |  | 1 | 0.69^**^ | 0.80^**^ | 0.73^**^ |  |  |  |
| Mn |  |  |  |  |  |  |  |  | 1 | 0.51^*^ |  | -0.46^*^ |  |  |
| pH |  |  |  |  |  |  |  |  |  | 1 | 0.62^**^ |  |  |  |
| TOC |  |  |  |  |  |  |  |  |  |  | 1 | 0.87^**^ | 0.50^*^ |  |
| GDP |  |  |  |  |  |  |  |  |  |  |  | 1 | 0.65^**^ |  |
| Population |  |  |  |  |  |  |  |  |  |  |  |  | 1 | 0.80^**^ |
| ALA |  |  |  |  |  |  |  |  |  |  |  |  |  | 1 |

ALA: Arable land amount;

*: Correlation is significant at the 0.05 level (2-tailed), *p* < 0.05;

**: Correlation is significant at the 0.01 level (2-tailed), *p* < 0.01.

**Caption of figures:**

Fig. S1. Land use of Yinma River Basin

Fig. S2. *CF* values for heavy metals at different sampling sites

Fig. S3. TOC of the sediment samples in Yinma River

Fig. S4. pH of the sediment samples in Yinma River


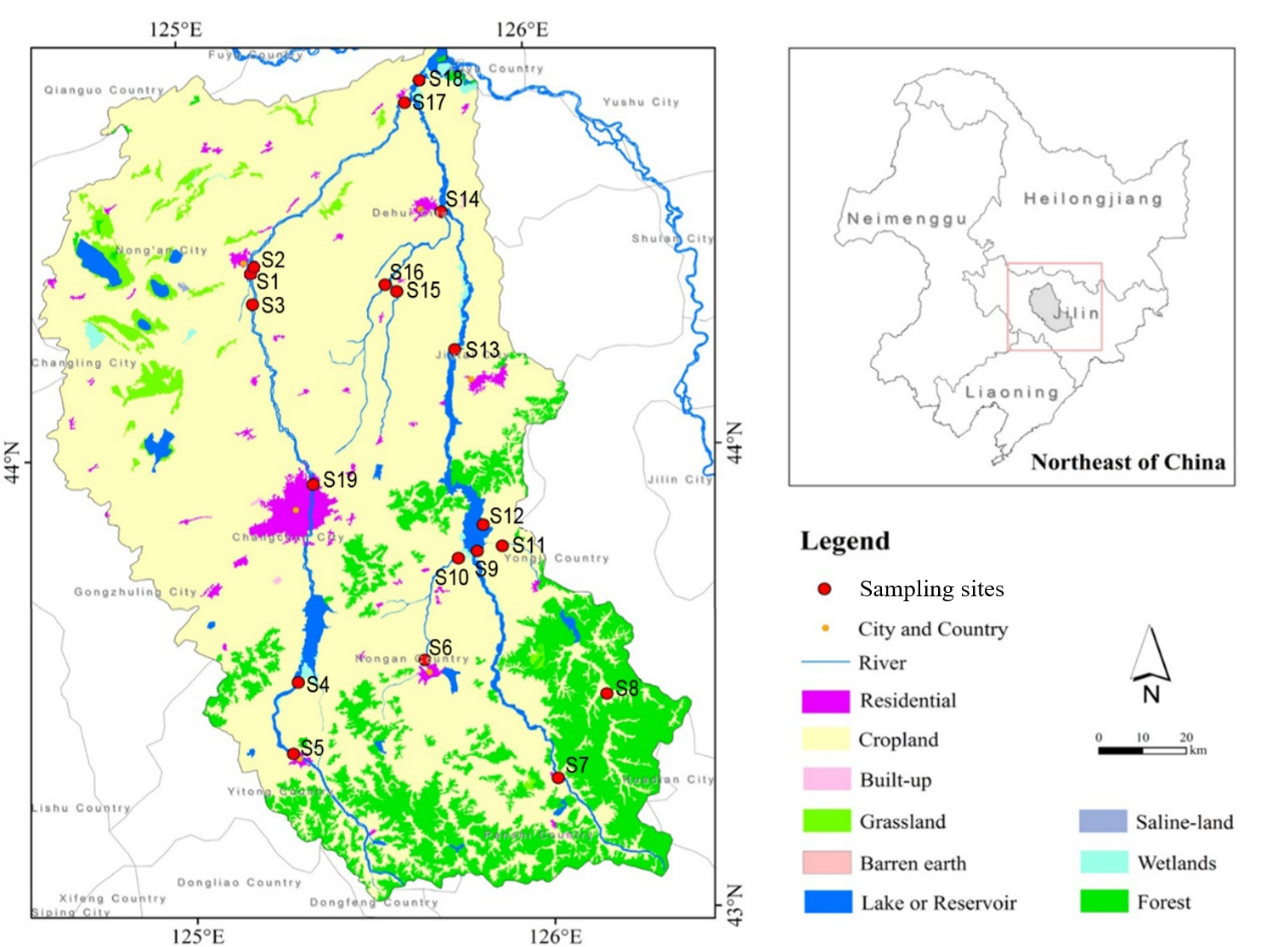


Fig. S1. Land use of Yinma River Basin





Fig. S2. *CF* values for heavy metals at different sampling sites


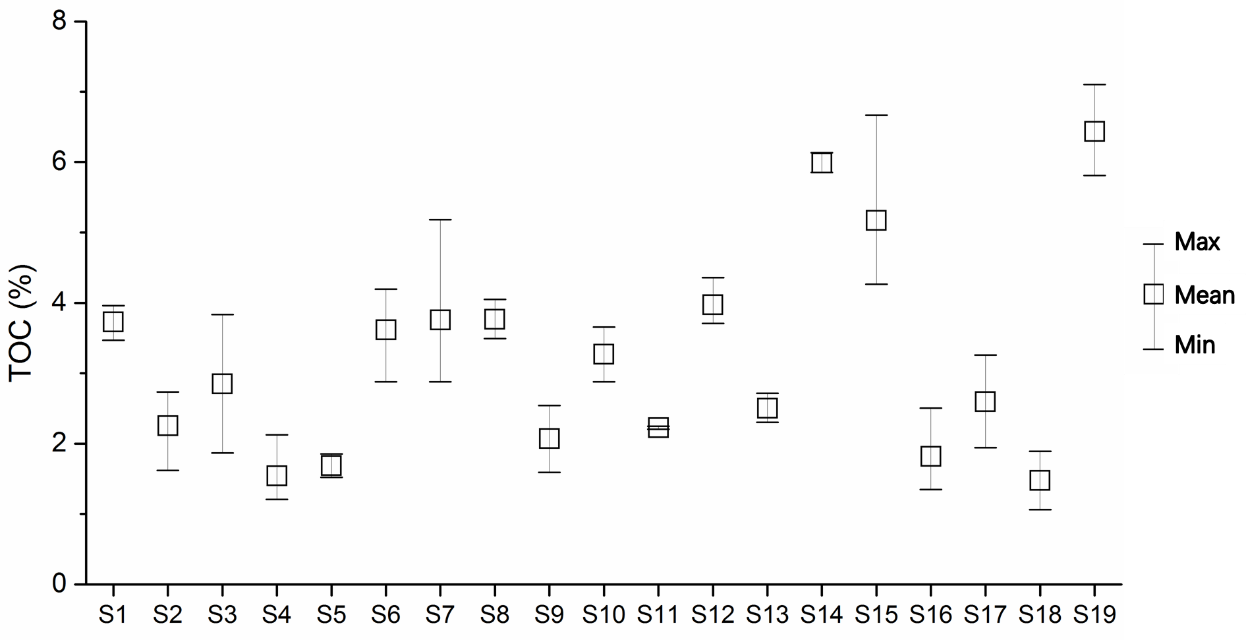


Fig. S3. TOC of the sediment samples in Yinma River


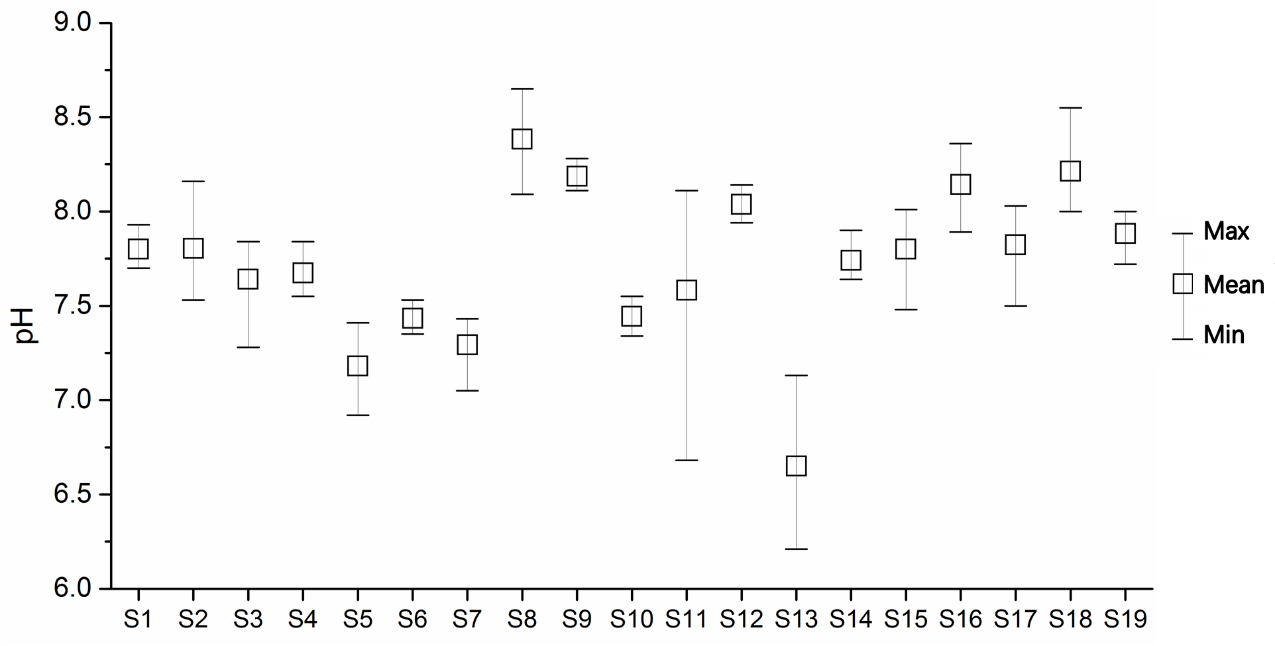


Fig. S4. pH of the sediment samples in Yinma River
